# A novel method for assessing postoperative femoral head reduction in developmental dysplasia of the hip

**DOI:** 10.1007/s11832-014-0600-5

**Published:** 2014-07-04

**Authors:** Anthony Cooper, Owain Evans, Farhan Ali, Mark Flowers

**Affiliations:** 1 Department of Orthopaedics, Sheffield Children’s Hospital, Western Bank, Sheffield, S10 2TH UK; 2 Trauma and Orthopaedics, Royal Manchester Children’s Hospital, Oxford Road, Manchester, UK

**Keywords:** Hip dysplasia, Anatomical variant, Radiological assessment, Developmental dysplasia hip, Computed tomography

## Abstract

**Purpose:**

Developmental dysplasia of the hip (DDH) affects approximately 1 % of live births. Dislocated hips require reduction and stabilisation in a spica cast, and reduction efficacy is assessed radiologically. Numerous measurements are used to ascertain the adequacy of reduction but can be inconsistent in evaluating femoral head position. This study describes the morphology of the developing acetabulum in DDH and validates a novel method to assess adequate reduction of the dysplastic hip following closed or open reduction.

**Methods:**

A retrospective review was performed of 66 consecutive patients undergoing reduction of hip dislocation over a 2-year period. Three independent reviewers evaluated postoperative CT scans to assess anterior-posterior (AP) displacement and modified Shenton’s line. Acetabular morphology was also assessed along with hip congruency using a described novel ‘posterior neck line’.

**Results:**

Dislocated hips were successfully identified using the posterior neck line with a sensitivity of 0.71 and specificity of 0.88 giving a negative predictive value of 0.97. The interobserver reliability of this technique was higher in comparison against both (AP) displacement and modified Shenton’s line.

**Conclusions:**

We have shown a novel approach in assessing the acetabular morphology of DDH and a novel technique to accurately confirm the reduction of dislocated hips following open or closed reduction.

## Introduction

Developmental dysplasia of the hip (DDH) is a common disorder affecting around one in 1,000 live births. For those patients with dislocated hips, closed or open procedures may be performed and the reduction held with hip spica casts. Standard radiographs have been superseded by single slice computer tomography (CT) to accurately assess the adequacy of postoperative reduction and identify hips that remain dislocated or redislocated [1, 2].

A number of measurements have been described to assess the adequacy of the reduction [3–5], but these have been shown to have limited value in predicting the rate of avascular necrosis (AVN) or the requirement for subsequent surgery [2]. The reliability and reproducibility of CT scan measurements have been shown to be lacking between raters [6]. With little consensus in the literature on adequate assessment of postoperative hip reduction, medial femoral head contact has been suggested as a marker of reduction [7]. This, however, relies on ossification of the femoral head, which is often delayed in DDH and prevents adequate assessment in those patients younger than six months.

The purpose of this study was to evaluate the morphology of dysplastic hips and to assess and validate the use of a new method to determine successful hip reduction postoperatively ensuring that the method was both reproducible and reliable.

## Methods

A retrospective review was made of 66 consecutive patients undergoing both closed and open reduction over a two-year period between August 2007 and August 2009. Postreduction CT scans were assessed by three independent reviewers (two paediatric orthopaedic registrars and a paediatric orthopaedic fellow) using specific criteria including acetabular morphology and the ‘posterior neck line’. The modified Shenton’s line previously described [5] was also assessed along with AP displacement. Minimal coaching was provided to the reviewers regarding the measurement techniques and all reviewers were given identical CT slice images generated per the standard protocol used in our paediatric radiology department.

**Fig. 1:**
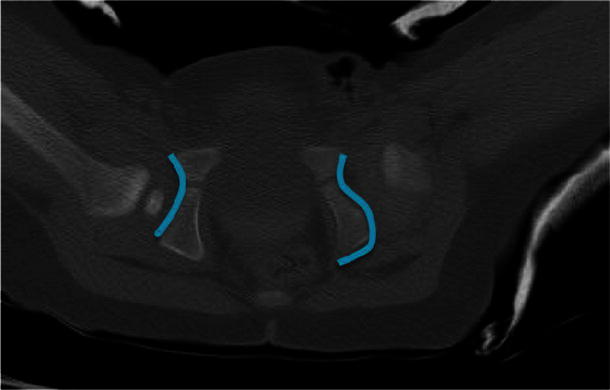
Postreduction single slice CT image showing the morphology of dysplasia

The acetabular morphology was described as either ‘C’ shaped, indicating the normal concavity in a hip without dysplasia or ‘S’ shaped, indicating a transition from the concavity of the acetabulum to the posterior wall convexity reproducibly seen in an abnormal dysplastic hip on the axial CT image as shown in Fig. 1.

**Fig. 2:**
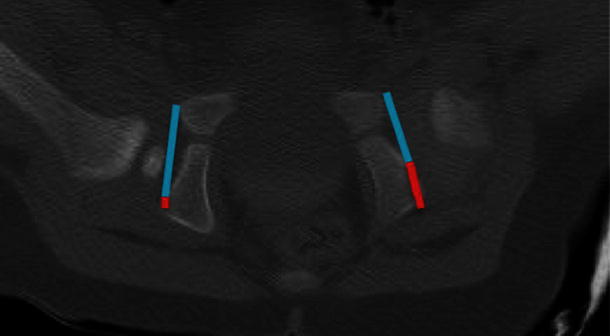
Postreduction single slice CT image highlighting the acetabular ratio

In order to quantify any apparent difference in acetabular morphology the acetabular ratio was measured as shown in Fig. 2. This was defined as the ratio of the acetabular diameter to the pelvic diameter on that side. The affected and non-affected side acetabular ratios were compared with each other to quantify the difference between the morphological ‘C’ and ‘S’ shaped acetabulum and confirm the anatomical abnormality.

**Fig. 3:**
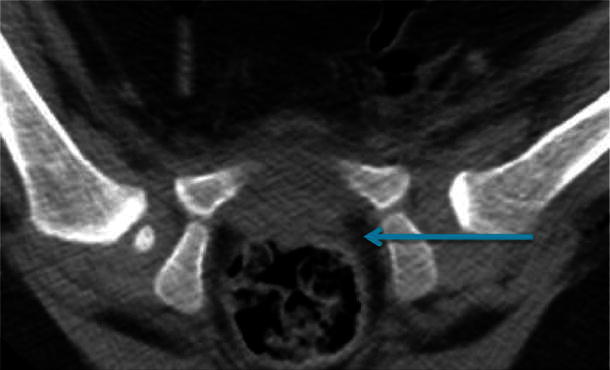
Postoperative single slice CT showing the posterior neck line in a reduced left hip

The posterior neck line is described as the continuation of a line from the posterior aspect of the femoral neck along the physeo-metaphyseal border of the greater trochanter of the proximal femur as shown in Fig. 3. This line is seen to traverse the acetabulum anterior to or at the point of transition of the concave acetabulum to the convex posterior wall in reduced hips but posterior to this transition point in dislocated hips.

The measurements were recorded independently and the affected side was blinded to the reviewers along with whether the hip was dislocated according to the senior author and a consultant paediatric radiologist who identified the dislocated hips independently. There was agreement in all cases. Data was entered into a standard spreadsheet and then analysed using SPSS 19 (IBM New York, New York City) and the interobserver error calculated.

## Results

Seventy-two hips were identified in a total of 66 patients (six bilateral). 38 cases followed closed reduction and 34 open reduction in 58 female and 14 male patients. The left hip was involved in 44 cases. The age for all reductions ranged from three to 53 months. The means for closed and open reductions are shown in Table 1.

**Table 1:** Mean ages for operative hip reduction

Procedure	Mean age (months)	Min age (months)	Max age (months)
All reductions	15.6	3	53
Closed reduction	9	3	24.6
Open reduction	22.9	9.1	53

Of all the affected hips an ‘S’ shaped acetabulum was described by all the reviewers, allowing correct identification of a dysplastic hip. All normal hips were described as ‘C’ shaped.

The mean acetabular ratio of affected hips was measured by the first author (APC) and found to be 0.63 with the mean index of unaffected hips at 0.75. Using the paired *T* test this was found to be a significant difference *P* < 0.001 (95 % CI 0.1–0.15) between the acetabular ratio in the unilateral cases between the affected and unaffected hips thus confirming the ‘S’ and ‘C’ variations in acetabular anatomy.

**Fig. 4:**
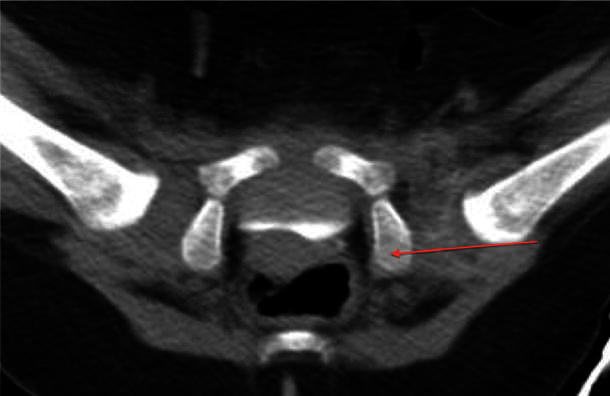
Postoperative single slice CT image with the posterior neck line revealing a dislocated left hip

Six hips were described as dislocated by the senior author and consultant radiologist and the accuracy of the posterior neck line measurement at identifying these dislocations (as measured by all three investigators) was calculated giving a mean sensitivity of 0.71 (range 0.4–1) and a mean specificity of 0.88 (range 0.85–0.98). This gave a mean negative predictive value of 0.97 (range 0.95–1) making this measurement effective in identifying those hips that were dislocated as shown in Fig. 4.

The interobserver reliability of the posterior neck line assessment was calculated using the Kappa coefficient of each reviewer to the other two reviewers and creating a mean Kappa value. Pearson’s coefficient was calculated in the same manner for the AP displacement and Shenton’s line assessment as a comparison, shown in Table 2. The Kappa coefficient was selected for the posterior neck line agreement as it is a measure of agreement of categorical values. Pearson’s correlation coefficient was used to assess agreement for AP displacement and Shenton’s line since the data is linear. The larger the number, the greater the correlation, 1 being complete agreement and 0 being no agreement.

**Table 2:** Interobserver correlation

Measure	Reviewer 1	Reviewer 2	Reviewer 3	Analysis
Posterior neck line	0.28	0.44	0.45	Kappa
AP displacement	0.12	0.16	0.27	Pearson’s
Shenton’s line	−0.01	0.04	0.33	Pearson’s

## Discussion

It is essential to confirm objectively the reduction of a hip following open or closed surgery. CT evaluation for this purpose is increasingly available, and, by minimising the number of CT slices obtained, the radiation dose is kept to a minimum. The radiation dose varies dependent on the age of a child. For a 1-year-old child the total radiation exposure is 1 mSv. This would include a scout film and 1–3 cuts. The typical radiation exposure risk to a 12 month child from this technique is considered to be “very low risk” compared with “negligible risk” for plain radiographs (National Radiological Protection Board). This method provides superior information compared with plain radiographs and allows accurate radiological assessment of the hips when stabilised in a spica cast [8]. The morphology of the acetabulum can also be visualized, thus,facilitating this assessment. There has, however, been previous conjecture regarding the reliability of measurements in assessing the congruency of the hip in DDH, and interobserver correlation has proved variable [2, 6].

The difficulty in assessing hip congruency may be due to the relatively young age groups that are assessed. Below 12 months, the ossification centre of the femoral head may not be present and is often eccentric. The bony landmarks of the pelvis are poorly defined in the younger patient allowing for difficult assessment of indices leading some groups to show poor reliability [3]. This has meant that magnetic resonance imaging (MRI) may be a superior modality in assessing the cartilaginous and soft tissue components of the developing hip [9]. MRI, however, is not readily available in all institutions and these patients require a general anaesthetic in order to obtain the necessary images.

On a single slice axial CT scan, sectioned through the tri-radiate cartilage, dysplastic hips typically feature a small concave acetabulum with a large ischial convexity posteriorly creating an ‘S’ shaped acetabulum that can be easily recognised and has not been previously described. The acetabular ratio between the normal and abnormal hips is significantly different (0.75 vs. 0.61) and further studies are recommended to determine the value at which the acetabular ratio becomes abnormal. We consider this to represent a poor modeling response in the ischial portion of the acetabulum as a result of the absence of normal loading from the (dislocated) femoral head.

**Fig. 5:**
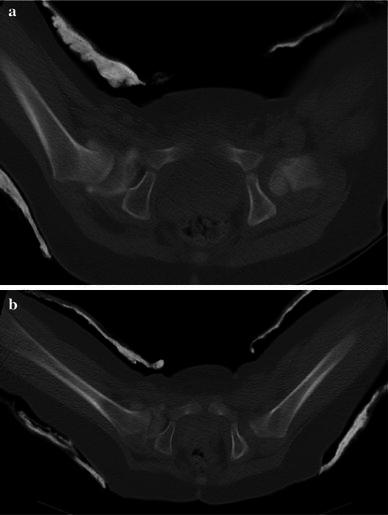
**a** Postreduction single slice CT image with hips in neutral position compared with the hips in a more abducted position **b** in the same patient following change of spica cast

The level at which the concavity turns to convexity in the affected hip offers a useful and important landmark with which to assess the reduction of the femoral head when measured along the posterior femoral neck. When the hip is located, the posterior neck line will meet the junction of the concavity (the posterior edge of the immature true acetabulum) and the convexity of the ischium within a range of abduction as seen in Fig. 5a and b. Hips are placed in the zone of Ramsay and the evident range of acceptable abduction demonstrated by the posterior neck line technique, therefore, supports the concept of that ‘safe’ zone.

The posterior neck line is a measurement that effectively identifies hip dislocation following surgery on single slice axial CT images. The interobserver correlation using the posterior neck line has been shown to be stronger than in both the modified Shenton’s line and the AP displacement, which are used only as a measure of displacement and not a definitive indicator of hip reduction. However, caution should be used when interpreting these statistical results, since one reviewer had a relatively low Kappa value, and it is not possible to directly compare the correlation coefficients of different measures.

The posterior neck line measurement offers a high negative predictive value, which is desirable to avoid undetected hip dislocation and its resultant detrimental sequelae.

## Summary

The posterior neck line is described as a new technique to confirm the adequacy of hip relocation on single slice postoperative axial CT scanning following open or closed surgery. We have demonstrated that the technique has superior reliability, and we suggest it should be considered as the preferred technique for assessment of hip relocation in those centres where postoperative CT scanning is routinely available.
